# Mechanical circulatory support devices and treatment strategies for right heart failure

**DOI:** 10.3389/fcvm.2022.951234

**Published:** 2022-09-23

**Authors:** Taiyo Kuroda, Chihiro Miyagi, Kiyotaka Fukamachi, Jamshid H. Karimov

**Affiliations:** ^1^Department of Biomedical Engineering, Lerner Research Institute, Cleveland Clinic, Cleveland, OH, United States; ^2^Department of Biomedical Engineering, Cleveland Clinic Lerner College of Medicine of Case Western Reserve University, Cleveland, OH, United States

**Keywords:** biventricular heart failure, right ventricular assist device, heart transplantation, ECMO, left ventricular assist device, mechanical circulatory support device

## Abstract

The importance of right heart failure (RHF) treatment is magnified over the years due to the increased risk of mortality. Additionally, the multifactorial origin and pathophysiological mechanisms of RHF render this clinical condition and the choices for appropriate therapeutic target strategies remain to be complex. The recent change in the United Network for Organ Sharing (UNOS) allocation criteria of heart transplant may have impacted for the number of left ventricular assist devices (LVADs), but LVADs still have been widely used to treat advanced heart failure, and 4.1 to 7.4% of LVAD patients require a right ventricular assist device (RVAD). In addition, patients admitted with primary left ventricular failure often need right ventricular support. Thus, there is unmet need for temporary or long-term support RVAD implantation exists. In RHF treatment with mechanical circulatory support (MCS) devices, the timing of the intervention and prediction of duration of the support play a major role in successful treatment and outcomes. In this review, we attempt to describe the prevalence and pathophysiological mechanisms of RHF origin, and provide an overview of existing treatment options, strategy and device choices for MCS treatment for RHF.

## Introduction

The number of heart failure (HF) patients reached more than 6 million in the U.S. in 2018 and is expected to rise to 8 million by 2030 (1). The overall incidence is increasing in Europe, as well (2–4). The same trend is being observed worldwide (5). Among this population, right HF (RHF) is associated with increased mortality (6, 7). Furthermore, the pathogenesis of RHF varies, and right ventricular (RV) function has a close interactive relationship with left ventricular (LV) function. For example, LV contraction generates approximately 30% of RV contraction energy, since the ventricles share the interventricular septum and pericardium (7, 8); on the other hand, RV dilatation may decrease the LV preload and ventricular elastance by shifting the interventricular septum and distensions of the pericardium. Therefore, the RHF management has been rather cumbersome due to absence of a standardized strategies for RHF particularly with use of MCS devices. Additionally, the absence of the dedicate durable RVAD device, less advanced stage RHF at the time of LVAD, variable rates or RV deterioration post implantation, surgical adaptation of the LVAD for RVAD use, and early stage biventricular failure adds up when it comes to the decision making process for optimal management strategy.

Currently, the indication for mechanical circulatory support (MCS) treatment for RHF is for patients who are refractory to medical or surgical therapy (8), however, the timing and the strategies for MCS treatment differ by the pathogenesis of the RHF and the duration of the support. Additionally, it is important to select MCS device with a solid understanding of the underlying mechanisms of the disease and therefore to determine the most beneficial device performance to achieve optimal degree of ventricular unloading.

## Right heart failure pathogenesis

### Isolated right ventricle injury pathogenesis

The pathogenesis of RHF can be divided into three major categories: isolated RV injury, pulmonary etiology, and the one secondary to LV failure (6). Additionally, there is the “after cardiac transplantation” condition and congenital heart disease condition; however, due to the complexity of these conditions, we have not included these conditions in this review.

The isolated RV injury pathogenesis, mainly associated with the decreased RV contractility, is one of the ways RHF develops. Some cases of acute inferior myocardial infarction (MI) are representative of isolated RV injury. Acute inferior MI patients with high-grade proximal occlusion of the right coronary artery tend to show severe RV ischemic dysfunction, which may result in higher in-hospital mortality (9). However, many patients show clinical improvements within 3 to 10 days, global RV performance recovers to almost normal levels within 3 to 12 months, and pure secondary unilateral RHF is rare (10, 11). A prospective study (*n* = 69) documented the long-term mortality among patients with RV MI after the first year was at an additional 2/year to 3%/year through 10th year (12).

### Pulmonary pathogenesis

Pulmonary pathogenesis, which caused by increased RV afterload inducing RV pressure overload and hypoxia, is a broad category. The representatives of this pathogenesis are pulmonary hypertension (PH) and acute pulmonary embolism (PE). Regarding PH, the world health organization (WHO) classification for PH and the European Society of Cardiology and European Respiratory Society guidelines for PH may help organize the topic (6, 13). In WHO classification, PH is classified as:

- Group 1: pulmonary arterial hypertension (PAH) (e.g., idiopathic, hereditary);
- Group 2: left heart disease (e.g., HF with preserved ejection fraction, HF with reduced ejection fraction);
- Group 3: lung disease (e.g., chronic obstructive pulmonary disease);
- Group 4: chronic thromboembolic disease (e.g., chronic thromboembolic PH);
- Group 5: miscellaneous (e.g., sarcoidosis, chronic hemolytic disorders).

It should be noted here that the most prevalent group is group 2 (68%), which is the group of PH due to left heart disease, such as systolic and diastolic dysfunction of LV (14). Therefore, strictly speaking, group 2 PH will be classified into left ventricular failure pathogenesis. The next prevalent group is group 5: miscellaneous (15%), Group 3: lung disease (9%), group 1: PAH (3%), and group 4: chronic thromboembolic disease (CTEPH) (2%) follows. A multi-center observational, prospective study (*n* = 2,635) showed a survival rate among PAH patients, which was 85 ± 1%, 68 ± 1%, 57 ± 1%, and 49 ± 1% at 1, 3, 5, and 7 years from diagnosis, respectively (15). The mortality among Group 2, 3, and 5 varies based on the disease and severity. Among CTEPH patients, a surgical treatment, pulmonary thromboendarterectomy, has shown to improve both short-term and long-term survival. In an international prospective study (*n* = 679) described that estimated survival at 1, 2, and 3 years was 93% (95% confidence interval [CI], 90–95), 91% (95% CI, 87–93), and 89% (95% CI, 86–92) in operated patients (*n* = 404), and only 88% (95% CI, 83–91), 79% (95% CI, 74–83), and 70% (95% CI, 64–76) in not-operated patients (*n* = 275).

### Left ventricular failure pathogenesis

As noted earlier, the RV function has a close interactive relationship with the LV function. As a result, the cause of the RHF in this pathogenesis could be decreased RV contractility and/or RV pressure overload. Cases are equally distributed in a wide variety of causes, such as myocarditis, LVAD support, ischemic disease, and mitral/aortic valvular heart disease; however, as long as this pathogenesis is secondary, the MCS for RHF is considered after or while receiving the LV treatment. Also noted earlier, this pathogenesis will include the patients with group 2 PH. Particular attention should be paid to the HF with preserved ejection fraction (HFpEF). The initial step should be made to treat RHF due to HFpEF after or while receiving the LV treatment; however, the treatment for HFpEF is still limited. Several efforts are undergoing in both medical and device-based treatment (16–18). Regarding the survival for HF with reduced ejection fraction (HFrEF), despite of remarkable advance in management of HFrEF, the 5-year survival after hospitalization remains poor (24.7%), and it was similar to HFpEF (24.3%) (19).

## Device descriptions

**Figure 1:**
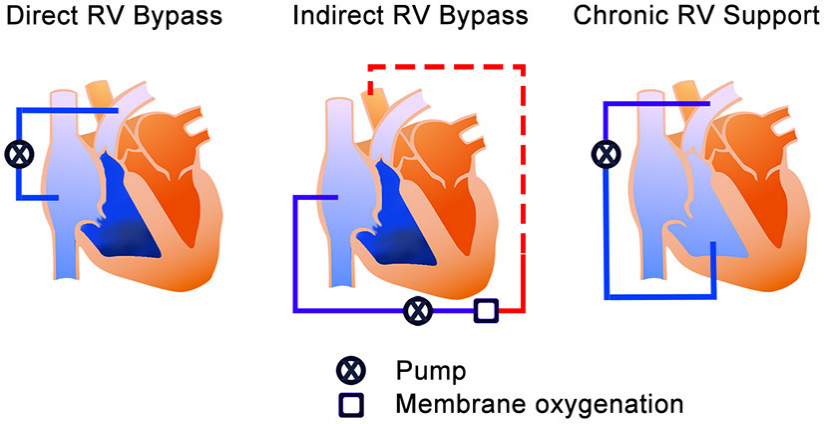
The treatment mechanisms of the MCS device for RHF. The blue color is un-oxygenated blood, and the red color is the oxygenated blood. Left—Direct RV bypass mechanism; takes blood from the systemic veins and pumps it to the pulmonary artery. Middle—Indirect RV bypass mechanism; takes the blood from systemic veins and provides systemic perfusion (red dash line) from the femoral artery. Right—RV support mechanism; takes blood from the RV and pumps it to the pulmonary artery. RV, right ventricle; MCS, mechanical circulatory support; RHF, right heart failure.

According to Kapur *et al*., the treatment mechanism of MCS devices for acute RHF can be divided into two categories; direct RV bypass and indirect RV bypass (20). The direct RV bypass devices, such as percutaneous temporal RV assist devices (RVADs), generally have the inflow in the inferior vena cava (IVC) or the right atrium (RA) and pump the blood to pulmonary artery (PA). On the other hand, the indirect RV bypass device, such as the veno-arterial extracorporeal membrane oxygenation (VA-ECMO), delivers blood from the systemic veins and provides oxygenated blood to systemic organs from the femoral artery access. Furthermore, we have extended Kapur's categories to include chronic RV support in which off-label use of the commercially implantable left ventricular assist device (LVAD) is used as RVAD. RVAD in this setup pumps blood through the graft anastomosed to PA. The difference from the previous two bypass groups is the implantability techniques and duration of support. Thus, it may be suitable for the long support duration in selected cases; however, surgical intervention will be needed. In this review, this option has been added as “chronic RV support” to explain the devices for both acute and chronic RHF (Figure 1). Additionally, for convenience of explanation, we included total artificial heart (TAH) in chronic RV support category.

### Direct RV bypass devices

#### Impella RP

**Figure 2:**
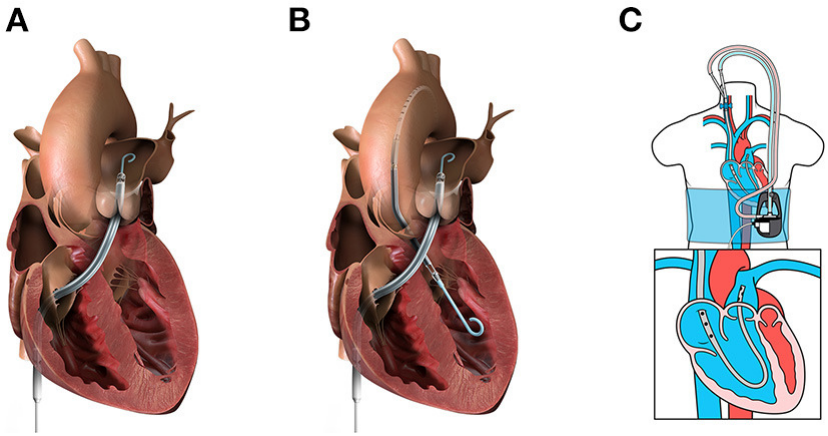
Percutaneous mechanical circulatory support devices for RHF. **(A)** Impella RP (image from Abiomed media kit, used with permission). **(B)** BiPella (image from Abiomed media kit, used with permission). **(C)** LifeSpark pump with ProtekDuo cannula (image from Livanova Investor Day 2021 presentation, used with permission).

The Impella RP^®^ (Abiomed, Danvers, MA, USA) is a U.S. FDA-approved, 22 Fr micro axial pump mounted on an 11 Fr catheter (Figure 2A). The pump is designed to provide up to 4.0 L/min at 33,000 rpm and support up to 14 days. The system is delivered *via* percutaneous femoral vein access, and the optimal pump inflow is designed to be positioned in the inferior vena cava and the pump outflow in the distal main PA, below the bifurcation to the right PA, which falls into the category of a direct RV bypass device. The device may be used in the temporary support duration with isolated RV failure pathogenesis and also secondarily to LV failure pathogenesis as a part of biventricular support.

The efficacy of the Impella RP was investigated in a prospective study, the RECOVER RIGHT study, in 2015 (21–25). The study consisted of two cohorts among 15 U.S. institutions: patients with RHF within 48 h post-LVAD implant (*n* = 31) and post-cardiotomy or post-MI patients with RHF (*n* = 29). The primary endpoint was survival at 30 days, hospital discharge post-device explant, or transition to the subsequent therapy, and was achieved in 73.3% of the study population. The total duration of device support in this study was ~ 3 days. The major adverse events at 30 days were major bleeding (60%) and hemolysis (13%). PA perforation occurred in one patient, which led to hemothorax, and was likely caused by the guidewire used during the device positioning. No PE was reported.

Recently, for severe biventricular support with suboptimal LV unloading using VA-ECMO, using two Impella pumps, known as BiPella (Figure 2B), can substitute V-A ECMO if oxygenation is not required. The BiPella therapy combines the LV Impella^®^ systems (5.0, CP, and 2.0) and Impella RP (26). A retrospective study among five U.S. hospitals (*n* = 20) reported that in-hospital mortality was 50% (27). In addition, non-survivors had higher PA resistance than survivors, which suggests that non-survivors might not be a biventricular failure but PH following the LV failure. Therefore, there may still be room for improvement in outcomes with attention to the pulmonary vascular load.

#### LifeSPARC pump with protekduo cannula

LifeSPARC Pump^®^ (LivaNova, Houston, TX, USA), formerly called TandemHeart, is an extracorporeal continuous-flow (CF) centrifugal flow pump with a magnetic pivot bearing (Figure 2C). The pump body priming volume is 16 mL and is designed to provide up to 4.5 L/min through the percutaneous catheter. With the development of the 29/31 Fr ProtekDuo dual-lumen cannula^®^ (LivaNova, Houston, TX, USA), the LifeSPARC Pump is often used with the ProtekDuo cannula percutaneously *via* the right internal jugular vein (25, 28). The ProtekDuo cannula contains two lumens: one lumen works as inflow and the other as outflow. The delivery technique is similar to the Swan-Ganz catheter insertion. The optimal positioning is the inflow in RA and outflow in main PA. LifeSPARC Pump and ProtekDuo cannula gained approval for support of up to 30 days by the European Medicines Agency and up to 6 days by the U.S. FDA (25).

The outcome of this system is mixed. In a report of 17 patients (12 patients had a durable LVAD in place) who went through the implantation with this system, during the mean length of support of 10.5 ± 6.5 days, 23% of patients were successfully weaned, 35% required conversion to either a surgical temporary extracorporeal RVAD (sRVAD) or durable implantable RVAD, and 41% did not survive (29). Another study reported that 27 LVAD patients received the system implantation, and device weaning occurred in 86% of patients, with 15% in-hospital mortality (30). Other studies also report similar good outcomes (31, 32).

The insertion without touching the groin region will be one of the advantages of this system. Although the duration of the support might have an impact, infection does not appear to be an issue. In addition, the insertion location enables another advantage for rehabilitation. Furthermore, this system is able to add a membrane oxygenator to the circuit if needed (32, 33). Interestingly, the TandemLung^®^ system (LivaNova, Houston, TX, USA) is developed to permit extracorporeal life support circuit to be wearable, and the system consists of LifeSPARC pump, ProtekDuo^®^ cannula, VoyagerVest^®^, and TandemLung^®^ (34).

#### Surgical extracorporeal RVAD

In LV failure pathogenesis, nearly 41% of RVAD implant occurred 0–2 days after LVAD implant, and 23.4% of RVAD implant occurred within 3–14 days (35). At the same time, successful RVAD weaning rates were reported as> 60%, with intermediate support duration of 13–17 days. Among those cases, sRVAD has been the standard procedure because the patients have fresh sternotomy incisions; therefore, access to the RA and PA may not be difficult. An outflow cannula of sRVAD is often surgically implanted directly *via* PA or anastomosed prosthetic vascular graft. The inflow cannula is implanted directly into the RA or *via* the femoral vein to the RA. The extracorporeal centrifugal pump, similar to the one used in ECMO, is the most commonly used in sRVAD, and the system is expected to provide the pump flow of approximately 4–5 L/min, working as a direct RV bypass system. This configuration is also used in post cardiotomy shock.

### Indirect RV bypass device

#### Veno-arterial extracorporeal membrane oxygenation (VA-ECMO)

ECMO therapy has been increasingly used, and the term became popular in the non-medical population during the COVID-19 pandemic. VA-ECMO is one of the widely used configurations of ECMO, which works as an indirect RV bypass mechanism for RHF. The system is effective at any point of the pathogenesis, but especially in the pulmonary etiology to avoid pressurizing the pulmonary vasculature. However, the flow from the VA-ECMO may increase the afterload for the heart, which can lead to pulmonary edema secondary to LV failure; therefore, clinicians must balance the pump flow (36).

Commonly, the peripheral cannulation *via* the femoral artery and femoral vein will be performed and will be connected to the membrane oxygenator and extracorporeal centrifugal pumps, such as CentriMag^®^ (Abbott, Abbott Park, IL, USA), Rotaflow II^®^ (Getinge, Göteborg, Sweden), CAPIOX^®^ (Terumo Cardiovascular, Ann Arbor, MI, USA), and MERA^®^ Centrifugal Blood Pump (Senko Medical Instrument, Tokyo, Japan) (6). Due to the incomplete full unloading of the ventricles, LV venting options may be needed (37, 38). Anticoagulation is recommended with an activated clotting time of 180–220 sec or a partial thromboplastin time of 65–90 sec (24). The advantage of this device is ease of placement, which allows it to serve as a bridge to a decision in an emergent clinical scenario (36). The system can provide circulatory support for up to 30 days.

The clinical outcome data of using VA-ECMO to isolate RV failure pathogenesis is limited to small cases studies (39–41); however, RV function seems to recover during the intermediate support period. (The clinical data that support using VA-ECMO is discussed in the previous section). In cases that are secondary to LV failure, the combination therapy of VA-ECMO and LV Impella, known as ECPELLA, has been increasing. A meta-analysis reported that short-term mortality among the ECPELLA cohort was 56.1%, which was better than VA-ECMO-alone therapy (63.7%), and occurrence of the major bleeding did not show a significant difference; however, hemolysis and renal replacement therapy were observed with a higher incidence of 36.8 and 51.6% than VA-ECMO alone (42). The report also noted that the size choice of the Impella device might have an impact on the rate of major bleeding.

### Chronic RV support devices

#### Dual HeartMate 3 device use

Due to the lack of long-term support for MCS, the durable implantable CF LVAD has become the off-label use for durable RVAD. In the U.S., Medtronic Inc. (Minneapolis, MN, USA) announced that it was withdrawing the HeartWare Ventricular Assist Device^®^ (HVAD) from the global market in 2021; therefore, HeartMate 3^®^ (HM3) (Abbott, Abbott Park, IL, USA) is the only U.S. Food and Drug Administration-(FDA) approved LVAD in 2022. HM3 is a CF centrifugal pump with full maglev bearing, displacing volume of 80 ml. For the anatomical limitation, the placement of the right pump may be a concern (43). In one report with 14 patients who received a dual HM3 implant as biventricular support configuration, the HM3 was implanted into the RA using felt spacers to decrease the intraluminal length of the inflow cannula (44). The pump pocket was made with polytetrafluoroethylene patches, as it protrudes into the right thoracic cavity. Moreover, due to the physiologic limitations, the RVAD must work in a below-designed afterload range; in other words, the pump must operate in a low pump speed range (43). As a result, pump thrombosis occurrence has been consistently reported at 36–37% (43, 45, 46). To avoid pump thrombosis, a modification to the outflow graft–making the graft diameter smaller–has been made to elevate the afterload of the RVAD (47).

In the same study of 14 patients who received dual HM3 implantation (44), five patients downsized the outflow graft, nine did not, and just one pump thrombosis was reported. The pump thrombosis occurred in the patient in whom both pumps were used as a TAH with excising both ventricles (unknown about graft downsizing).

In another study with dual HM3 implantation, 12 patients underwent surgery for the bridge to transplantation (BTT). The RVAD was implanted to the RA using the spacer and was wrapped with a Gore-Tex^®^ (W.L. Gore, Flagstaff, AZ) Soft Tissue Patch and placed in the right pleural space. Graft downsizing was done in three cases, and pump thrombosis was observed in three patients (48). The relationship between pump thrombosis and graft downsizing was unknown; however, in two of the three cases, the pump thrombosis was likely dislodgement of an intracardiac thrombus and ingestion into the pump. Among these cases, the incidence of pump thrombosis seems to be lower than the previous reported series.

The authors also suggested that the extremely low thrombosis risk of the HM3 as an LVAD may explain this phenomenon (48). The major difference between the two pumps is their operation range, which is the result of pump-bearing design features. The investigation for the thrombogenicity among two pumps working at low range speed may provide the justification for their suggestion. The clinical outcome for dual HM3 varied among two studies; the survival at 18 months was 54.6–91.7%.

#### Dual Berlin Heart EXCOR

The Berlin Heart EXCOR^®^ (Berlin Heart, GmbH, Berlin, Germany) is a paracorporeal pneumatic-drive pulsatile device with a lineup of different pump sizes that cover pediatric to adult (10, 15, 25, 30, 50, 60, and 80 ml) with different valves (tri-leaflet polyurethane or bileaflet carbon valves). For the variety of the pump sizes, dual Excor has been used as a BVAD, especially in the pediatric population (49–51). The cannula implantation for the right-side pump is made *via* the RA for the inflow and the PA for the outflow. The overall survival at 1 year was approximately 40–83% at 1 year and 75% at 5 years for the adult (49, 52). Mortality among the pediatric patients ranged from 6 to 39%, and the transplantation rate from 37 to 73% (53).

#### SynCardia total artificial heart

**Figure 3:**
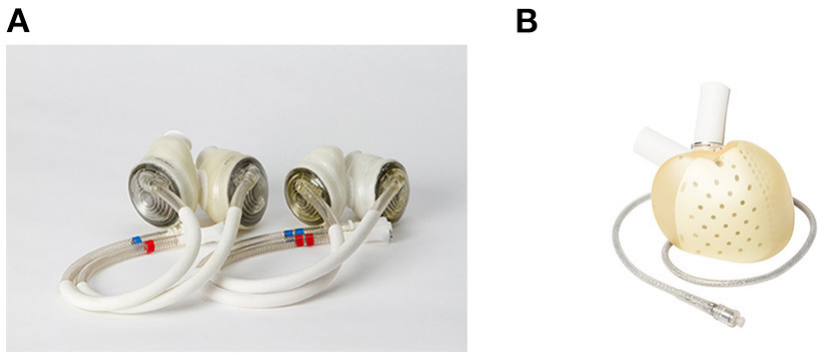
Current clinically available total artificial hearts. **(A)** SynCardia Total Artificial Heart 50 mL and 70 mL (55), used with permission). **(B)** Aeson Total Artificial Heart (used with permission by Carmat SA).

The SynCardia TAH^®^ (SynCardia Systems, LLC, Tucson, Arizona, USA) is a pneumatically driven pulsatile TAH with independent ventricles that are capable of providing a flow of more than 9 L/min (54) (Figure 3A). In addition, the SynCardia is a U.S. FDA-approved TAH indicated for use as a BTT in biventricular HF patients. Both the 50 ml and 70 ml size pumps are approved as BTT in the U.S., Europe, and Canada. Furthermore, in 2022, a 70 ml pump is undergoing an FDA clinical trial for destination therapy approval.

The clinical data supporting the SynCardia TAH, reported from an international registry, that the mortality on the transplantation waitlist was 7.4% for the 433 patients who underwent BTT therapy with TAH, and 87% of the patients reached heart transplant (HT) (56). Additionally, most of the patients were INTERMACS profile 1 (43%) or profile 2 (37%), and the risk factors for RHF were observed in 82% of patients (57).

#### Aeson total artificial heart

The Aeson^®^ TAH (Carmat, Vélizy-Villacoublay, France) is a pulsatile TAH that is electro-hydraulically driven (Figure 3B). The pump consists mainly of four biological valves and two ventricle chambers with a membrane that separates the chambers for the blood and actuator fluid (58). For the clinical data, results from a pilot study are available. Between 2013 and 2015, four patients were implanted Aeson TAH (59). The support durations were 74, 270, 254, and 20 days, and the causes of death were: two device-related (details unknown), one respiratory failure, and one multi-organ failure. Two patients were able to discharge home. In 2021, a pivotal study was ongoing in Europe, and the pump was approved by the U.S. FDA for the conduction of an early feasibility study (60).

## Right ventricle MCS device selection

### Devices for isolated right ventricle injury pathogenesis

Table 1 summarizes the current strategy for each HF pathogenesis and RV support duration. The top row of the table consist of three pathogenesis explained in previous section and suitable treatment mechanism of MCS devices. For isolated RV injury pathogenesis, the support duration may be short-term as described in the previous section, therefore, temporary use devices may fit the strategy in selected cases. Either direct or indirect RV bypass of the treatment mechanism may be considered suitable option, unless the pulmonary resistance is within acceptable boundaries. The durable RV support mechanism should involve the use of MCS device capable to deliver full cardiac output and suitable for long term support. Thus, VA-ECMO, Impella RP, and LifeSPARC pump with ProtekDuo cannula system may be a choice.

**Table 1:** Classification of the current mechanical circulatory support devices for RHF based on pathogenesis and the support duration.

	**Isolated RV failure** **Direct RV bypass** **Indirect** **RV bypass**	**Pulmonary etiology** **Indirect RV bypass**	**Secondary to LV failure** **Direct RV bypass** **Indirect RV bypass** **Chronic RV support**
**Temporary support** ( ≤ 14 days)	• VA-ECMO • Impella RP • LifeSPARC + ProtekDuo	• VA-ECMO	• VA-ECMO or ECPELLA • Impella RP or BiPella • HM3 (Left) + Impella RP + sRVAD + LifeSPARC + ProtekDuo
**Intermediate support** (≤ 30 days)		• VA-ECMO	• HM3 (Left) + Impella RP + sRVAD + LifeSPARC + ProtekDuo
**Long-term support** (≥1 year)			• HM3 + HM3 • EXCOR + EXCOR • SynCardia TAH • Aeson TAH

RV, right ventricle; LV, left ventricle; V-A ECMO, veno-arterial extracorporeal membrane oxygenation; HM3, HeartMate 3; sRVAD, surgical right ventricular assist device; TAH, Total artificial heart; Black dot marker defines it as an individual item, and plus marker represents the combination use with black dot item; ECPELLA, the combination use of Impella (LV support) and VA-ECMO (RV support). BiPella, the combination of Impella 2.0/CP/5.0 (LV support) and Impella RP (RV support).

In the rare case of isolated RV failure, such as the case of severe arrhythmogenic right ventricular cardiomyopathy, which requires RV long-term support, single report and study showed the feasibility of off-label use of HM3 and HVAD; however, the small number of patient remains to be insufficient to discuss the outcomes (61, 62).

### Devices for pulmonary pathogenesis

For pulmonary pathogenesis, among group 1, 3, 4, and 5 PH patients, acute RHF cases can happen at the time of initial PH presentation or acute on chronic situation (63). The balloon atrial septostomy (BAS) is the most commonly performed as palliation for refractory PH and progression to RHF (64). A meta-analysis revealed that BAS showed relatively high postprocedural and short-term survival; however, the long-term survival was less impressive, suggesting the bridging role for BAS (65). There is another option to use MCS for this bridging situation. In the MCS option, the treatment may have to bypass the lung, therefore, a use of VA-ECMO may be considered. The concept of MCS therapy is bridge to recovery or bridge to transplantation. A retrospective (*n* = 6) study showed that three out of four PAH patients who underwent bridge to recovery therapy successfully survived to VA-ECMO decannulation (mean support duration, 12 ± 7 days) (66). In addition, several report demonstrated that awake VA-ECMO therapy for bridge to transplantation concept was feasible, even for several weeks support (67–69). Besides, a case report described that a RHF due to PAH patient (40 years of PAH history), who received staged direct RV bypass device (ProtekDuo cannula with CentriMag pump) and chronic RV support (HVAD on right atrium) therapies, showed no pulmonary hemorrhage, even patient's systolic PA pressure was around 100 to 120 mm Hg (70); however, successful long-term use of direct RV bypass devices for PAH has not been reported yet.

Acute PE and some of PH may need to be treated as acute RVF. For example, a post-operative period in CTEPH (group 4), and acute respiratory distress syndrome (ARDS) (group 3). The most suitable treatment option is indirect RV bypass with VA-ECMO. The 2019 European Society of Cardiology guidelines for the management of acute PE defined high-risk PE patients as having hemodynamic instability, PE severity index class III-V or simplified PE severity index ≥ 1, RV dysfunction on transthoracic echocardiogram or computed tomography pulmonary angiography, and elevated cardiac troponin levels. Furthermore, the guidelines suggest VA-ECMO therapy as class IIb, evidence level of C, in combination with surgical embolectomy or catheter-directed treatment and use of rapid short-term support (71, 72). One multicenter study in Europe found that overall 30-day mortality with ECMO-alone therapy was 77.7% and suggested using ECMO as a complement to surgical embolectomy (73).

Conversely, a group from China reported in its subgroup analysis that the earlier ECMO treatment was associated with lower in-hospital mortality and significant overall survival (74). In case cardiac function improves while severe respiratory failure remains, the reconfiguration to veno-arterio-venous ECMO is reported effective (75, 76). For chronic thromboembolic PH post-operative ECMO is recommended as the standard of care due to the reperfusion edema in the early post-operative period (77, 78).

The importance of RHF to ARDS has been magnified by the COVID-19 pandemic (79–82). A retrospective analysis (*n* = 39) reported that patients who received percutaneous RVAD with ECMO therapy had significantly lower in-hospital and 30-day mortality than patients treated with invasive mechanical ventilation only (83). Theoretically, a direct RV bypass device for pulmonary pathogenesis may over-pressurize the pulmonary vasculature and may cause pulmonary hemorrhage (84). In spite of this, pulmonary hemorrhage was reported in 12.5% of patients, and no statistic difference was achieved (83).

### Devices for left ventricular failure pathogenesis

The treatment strategy for the LV failure pathogenesis should be made with a concern for each ventricle support duration. Also, the treatment mechanism can be chosen from either of direct RV bypass, indirect RV bypass, or chronic RV support, based on RV support duration. Regarding temporary to intermediate LV support, the representative cases may be cardiogenic shock, the post cardiotomy shock, and acute on chronic HF manifesting hemodynamic instability. Among those cases, the importance of trans-femoral or trans-axillary/trans-aortic percutaneous LVADs has been increasing (85, 86). If the symptom of RHF remains with enough LV unloading including LV Impella, the combination use of temporary MCS for RHF should be considered, because of its ease of implantation and explantation (20, 87). Therefore, Impella RP or LifeSPARC pump with ProtekDuo system may be an option for the right side. If patients manifest high pulmonary vascular resistance and a need for oxygenation, VA-ECMO device may be the choice. If only decreased RV contractility is observed, a direct RV bypass device may be suitable; however, a strategy in which direct RV bypass is connected to a membrane oxygenator may be suitable. Besides, the operators must concern which access (e.g., left or right, jugular or femoral) to cannulate the devices for building up the treatment strategy.

Regarding the long-term LV support for representative cases of durable LVAD implantation, RHF complicates 10% to 40% of LVAD implants (88, 89). LVADs will increase the incidence of RHF because they may shift the interventricular septum to the left and may decrease the septal contribution to RV contraction (90, 91). Furthermore, according to the 12th Interagency Registry for Mechanically Assisted Circulatory Support (INTERMACS) Report, 26,688 patients (96%) received isolated CF LVAD therapy, and 1,136 patients received a CF biventricular support device (BVAD) therapy, of whom 91.3% were supported with temporary CF RVADs (92).

In HM3 pivotal and post-pivotal trial study report, RHF was seen in 34.2–37.4% of patients, and 4.1–7.4% of patients required an RVAD implant (35). Notably, 40.6% of RVAD implants were performed within 2 days, and 23.4% of implants were performed between 3 and 14 days. Furthermore, the Mechanical Circulatory Support Academic Research Consortium divided the RHF after LVAD implantation into three groups:

- Early acute RHF, which is defined by the need for implantation of a temporary or durable RVAD before the patient leaves the operating room;
- Early post-implant RHF, which needs implantation of RVADs within 30 days following LVAD implantation or failure to wean from inotropic, vasopressor, or inhaled nitric oxide within 14 days following LVAD implantation or having to initiate these supports within 30 days;
- Lastly, late RHF, which need implantation of an RVAD more than 30 days after an LVAD or hospitalization with intravenous diuretics or inotropic support that occurs more than 30 days post-implant (93).

To summarize, approximately 40% of RHF after LVAD implantation patients may fall into early acute RHF, and 23% of RHF patients will present early post-implant RHF. However, the predictor for the RHF following LVAD implant still needs to be explored. In HM3 pivotal and post-pivotal studies, intra-aortic balloon pump use (odds ratio [OR]: 1.84), destination therapy (OR: 1.69), INTERMACS profiles 1–2 (OR: 1.60), and estimated glomerular filtration rate > 10 mL/min/1.73 m^2^ (OR: 0.9) were all reported as predictors for the RHF requiring RVAD (35).

The RV MCS device selection during long-term LV support may largely depend on the duration of the RV support. Unplanned temporary RVAD use was reported with successful RVAD weaning rates of over 60% with a median support duration of 13–17 days (25, 94–96). Therefore, short-duration RV MCS devices may be suitable for this strategy in selected cases. The treatment mechanism will be either direct or indirect RV bypass, which has more advantages with regard to conditions of pulmonary function and performance; however, there has been a single report of using a direct RV bypass device connected to a membrane oxygenator and aiming for early extubation; thus, pulmonary vascular resistance and removability may be the main concern (97).

As for long-term biventricular support, as discussed, the situation is rare, but it does exist. The options are limited due to the lack of durable MCS devices designed explicitly for RVAD. Currently, these options are: the off-label implantation of CF LVAD as an RVAD (Dual HM3); a paracorporeal pulsatile ventricular assist device as a biventricular support configuration (Dual Berlin Heart EXCOR); and TAH (SynCardia TAH or Aeson TAH). For the timing of RVAD intervention, severe late RHF, which is defined by the requirement for an RVAD at months 3–12, is very rare (98). However, recent data have been reported from Japan suggesting that late RHF is related to cardiac cachexia at HT, and increases the risk of infections within 6 months of HT (99). Thus, the timing of the intervention for late RVAD may change in the future with a prognosis of a specifically designed durable implantable RVAD.

As for the predictors for RHF, several research efforts are ongoing. The PA pulsatility index predicted the early RHF with a cutoff value of 2.0 (area under the curve, 0.77; sensitivity, 74%; specificity, 67%) (100). The RV global longitudinal strain predicted the early acute and post-implant RHF with a cutoff value of −9.7% (area under the curve, 0.86; sensitivity, 89%; specificity, 78%) (101).

## Future perspectives

**Figure 4:**
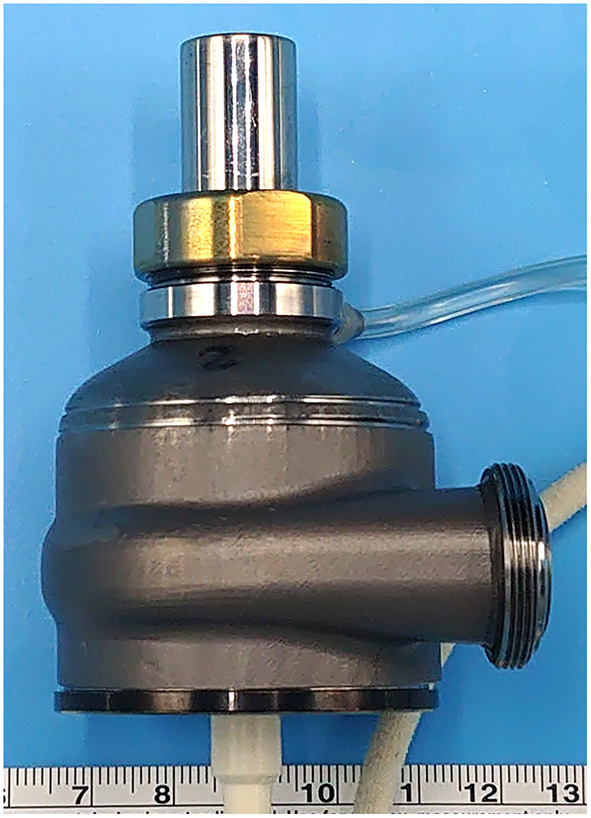
Cleveland Clinic Universal Ventricular Assist Device.

There seems to be major three work fields that should have explored. First, the development of specifically designed, durable RVAD may be required. The specifically designed ventricular assist device that is able to operate on both sides of the heart, such as the Cleveland Clinic Universal Ventricular Assist Device (UVAD, Figure 4), is seen as a promising, universal solution able to cover the hemodynamic needs of the broad HF patient population (102, 103). The UVAD is a hybrid of magnetically and hydrodynamically levitated centrifugal pumps, and is an innovative apparatus with a wide operating range, automatic regurgitant-flow shut-off, and pulse augmentation features (104). The unique design architecture of the UVAD permits accommodation of various hemodynamic profiles. The flexible operating range allows the pump to be used in a broad range of hemodynamic conditions, so it can be used on both right and left ventricular/atrium. Additionally, the automatic regurgitant-flow shut-off feature provides the feasibility for non-invasive pump-off tests, which plays an important role for RVAD weaning (105). The device is currently undergoing engineering optimization, and the developers are also focusing on addressing the need of dual-device operation using a single controller, for biventricular failure application as BVAD support.

Second, mobility of percutaneous RVAD must be improved. Currently, Impella RP is inserted from femoral vein because of the inlet of the cannula is designed to place in IVC and 11 Fr sheath (15 Fr outer diameter) stays at cannulation site, therefore, not suitable for rehabilitation. As for ProtekDuo cannula, 29 Fr cannula is inserted from right jugular vein; however, the large-bore cannula may still limit patients' outcome.

Third, the oxygenation support with RVAD may be interesting field to explore. RVAD circuit connecting to membrane oxygenator, known as Oxy-RVAD configuration, has been reported for use in the patient waiting for lung transplant (106). TandemLung^®^ system is applicable for this category.

## Discussion

With an increasing incidence of RHF in the last decade, the therapeutic and device options for this pathology remain unmet. While previously underestimated, the RV has triggered interest with the scope of better understanding of the underlying disease pathogenesis in order to provide potential therapeutic options and device solutions. With ongoing effort amongst multiple engineering and clinical groups, there has been a substantial spike in research interest and development of unique device-based target therapies.

The device selection for the shorter support duration seemed to have progressed to less invasive. To provide less invasive MCS therapy for RHF, the prediction for the support duration increases its importance. In the LV failure pathogenesis, more than 98% of patients received their LVAD therapy with centrifugal pumps in 2020, and more than 83% of patients were implanted HM3 (92). This trend is expected to accelerate in 2022 because the centrifugal pump with hybrid levitation is no longer available in the U.S. Therefore, most patients who present with RHF with LV failure pathogenesis will have HM3 implanted and will have a fresh sternotomy incision.

For this reason, sRVAD has been the gold standard for temporary- and intermediate-support for RHF. However, the effort to implant the HM3 with minimally invasive surgery has begun (107) and will have an impact on the current strategy because the ease of access to RA and PA will be unknown. Also, the less invasive method may be another key for the MCS therapy for RHF. If it is less invasive, the hurdle for making the decision to use MCS may lower, and intervention may begin earlier. The devices that will have the greatest advantage may be the percutaneous RVADs (pRVADs). Regarding the survival outcomes between the pRVAD and sRVAD groups, one study reported that there was no significant difference in 30-day mortality, 1-year survival, or 2-year survival, but the length of intensive care unit stay was significantly shorter in the pRVAD group (21 days vs. 34 days, *p* = 0.01) (108), however, data still remain scarce.

Regarding percutaneous MCS, the combination of Impella devices to support both ventricles has been increasing. The most typical examples are ECPELLA and BiPella. The difference between these treatment methods is whether to use Impella RP or VA-ECMO for the RV support. A meta-analysis showed that there was no significant difference between the ECPELLA cohort and BiPella cohort in mortality (*p* = 0.93) (109). Additionally, there was no significant difference in adverse events, major bleeding, hemolysis, and limb ischemia; however, the authors further prospective studies due to small sample sizes and the lack of hemodynamic data.

For long-term RVAD support, the currently available option is mainly limited to HM3 off-label use. The most concerning issue with this treatment is RVAD pump thrombosis. Regarding the necessity of outflow graft downsizing, one study operated the HM3 RVAD with a mean RVAD speed of 4,991 rpm and mean RVAD flow of 4.3 L/min (48). It was consistent with the pressure head curve of HM3 (110); if 4–5 L/min flow was demanded, 4,000–5,000 rpm pump speed would create a pump pressure rise of approximately 30–60 mm Hg, which is the pressure difference that may not need the graft downsizing. As a result, pump thrombosis was rarely reported. In addition, the HM3 has an artificial pulse mode feature, which improves the pump washout, and it will start of 4,000 rpm. Therefore, if the demanding flow is below 4 L/min, graft downsizing may be needed to operate the pump with increased speed.

Moreover, in this review, we discussed about one-to-one correspondence treatment strategy for each pathogenesis; however, in the real clinical world, the most of the patients are expected to have multiple pathogenesis. For example, the patient in acute exacerbation of American College of Cardiology/American Heart Association stage C heart failure with comorbidity of severe chronic obstructive pulmonary disease, may not be a rare case. Therefore, there may be a situation that LV is not bad as receiving LVAD therapy but RV requires MCS device, to overcome the acute decompensation. For these patients, applying the algorithm for RV MCS device us may be helpful (20).

Lastly, we did not include the RHF after HT because of the complexity of the mechanism and pathogenesis; however, the importance of MCS therapy for RHF is increasing. Moreover, since the first successful human HT was reported in 1967 in South Africa, more than 120,000 patients have received HT therapy (111), and the number who receive second and third HT transplants is growing (112). For those patients, each surgery becomes higher risk, and the risk of graft failure also increases. The need for MCS devices, including the TAH, may increase.

## Author contributions

TK: manuscript preparation. CM: critical manuscript review. KF: critical revision of article. JK: critical revision of article, approval of article. All authors contributed to the article and approved the submitted version.

## Funding

The Universal Ventricular Assist Device Program is supported by federal funding from the National Heart, Lung, and Blood Institute under grant 5R21HL133871 (to KF).

## Conflict of interest

Author KF is a co-inventor of Universal Assist Device. The remaining authors declare that the research was conducted in the absence of any commercial or financial relationships that could be construed as a potential conflict of interest. The reviewer MS declared a past co-authorship with one of the authors CM, KF, and JK to the handling Editor.

## Publisher's note

All claims expressed in this article are solely those of the authors and do not necessarily represent those of their affiliated organizations, or those of the publisher, the editors and the reviewers. Any product that may be evaluated in this article, or claim that may be made by its manufacturer, is not guaranteed or endorsed by the publisher.
